# Molecular Modeling of the Catalytic Domain of CyaA Deepened the Knowledge of Its Functional Dynamics

**DOI:** 10.3390/toxins9070199

**Published:** 2017-06-26

**Authors:** Thérèse E Malliavin

**Affiliations:** Institut Pasteur and CNRS UMR 3528, Unité de Bioinformatique Structurale, 28, rue du Dr Roux, F-75015 Paris, France; therese.malliavin@pasteur.fr

**Keywords:** *Bordetella pertussis*, adenyl cyclase, molecular dynamics simulation, enhanced sampling, virtual screening, thiophen ureoacids

## Abstract

Although CyaA has been studied for over three decades and revealed itself to be a very good prototype for developing various biotechnological applications, only a little is known about its functional dynamics and about the conformational landscape of this protein. Molecular dynamics simulations helped to clarify the view on these points in the following way. First, the model of interaction between AC and calmodulin (CaM) has evolved from an interaction centered on the surface between C-CaM hydrophobic patch and the α helix H of AC, to a more balanced view, in which the C-terminal tail of AC along with the C-CaM Calcium loops play an important role. This role has been confirmed by the reduction of the affinity of AC for calmodulin in the presence of R338, D360 and N347 mutations. In addition, enhanced sampling studies have permitted to propose a representation of the conformational space for the isolated AC. It remains to refine this representation using structural low resolution information measured on the inactive state of AC. Finally, due to a virtual screening study on another adenyl cyclase from *Bacillus anthracis*, weak inhibitors of AC have been discovered.

## 1. Introduction

The adenyl cyclase toxins are encountered in several organisms. Up to now, three of these toxins have been studied at the molecular level: the protein Edema factor (EF) from *Bacillus anthracis*, the protein CyaA from *Bordetella pertussis* and the protein ExoY from *Pseudomonas aeruginosa*. Among these three proteins, two of them, EF and CyaA, were successfully investigated by high-resolution X-ray crystallography [1,2]. Both proteins are activated as adenyl cyclase by interaction with the ubiquitous protein calmodulin, present in the cytoplasm of the host cell. The toxic protein enters the attacked cell in an inactivated form, gets activated by association with endogenous calmodulin (CaM), and triggers overproduction of cyclic adenosine monophosphate (cAMP), which in high concentration perturbs the cell signaling system, making its immune response inefficient. Several AC domains are only partially structured or unstructured [3,4], and the influence of the disorder within RTX (Repeat in ToXin) to the uptake and secretion of AC through the bacterial secretion machinery has been investigated [3,5,6,7]. The catalytic domain (AC) of CyaA is activated by its interaction with calmodulin, which induces a reorganization of the catalytic site, then able to overproduce cAMP. The X-ray crystallographic structure of AC has been determined [2] in complex with the C-terminal lobe of calmodulin (C-CaM).

CyaA has been studied over three decades in the Unit “Biochimie des Interactions Macromoléculaires”, and this protein revealed itself as a very good prototype for developing various biotechnological applications [8,9]. Paradoxically, little is known about its functional dynamics and about the conformational landscape of this protein. The appearance of its high-resolution structure [2] provided the occasion to start a molecular modeling study. One motivation for such a theoretical study is the possibility of a finer tuning of the AC dynamical properties, increasing the range of possible biotechnological applications. Another motivation is the discovery of new inhibitor families [10] able to fight against the whooping cough, whose *Bordetella pertussis* is the agent. In the context of increasing resistance of *Bordetella pertussis* to antibiotics [10,11], this search of inhibitors is relevant.

The present article is devoted to a review of the molecular modeling studies conducted on the AC domain of CyaA over the past. First, the knowledge on AC functional dynamics at the beginning of molecular modeling studies is presented. The evolution of the functional dynamics model following the publication of various molecular modeling studies is then described. The review is divided into three parts, corresponding to the three main axes of the AC study: (i) interaction between AC and calmodulin; (ii) conformational landscape of the inactive state of AC; (iii) inhibition of the AC activity.

Several molecular modeling techniques were used to obtain the results reviewed here. All of them are based on a classical empirical modeling of the protein structures, in which the electronic and nuclei parts of the energy are separated, the nuclei being modeled as rigid spheres, and the electronic cloud being modeled implicitly by empirical functions describing the effect of this cloud on the nuclei: for example, the effect of chemical bonds is usually modeled using a string set-up between the bonded atoms. Based on this empirical energy potential, the resolution of Newton equations of motions permits recording of molecular dynamics (MD) trajectories.More sophisticated schemes of molecular dynamics allows for enhancing the sampling of conformational space: for more information about them, see [12,13,14]. During enhanced sampling simulations, the system is considered to evolve in a multidimensional landscape, in which the regions of local minima are valleys and are described as basins. The experimental structure, used as a starting point of simulation, corresponds most often to a basin of low energy.

**Figure 1:**
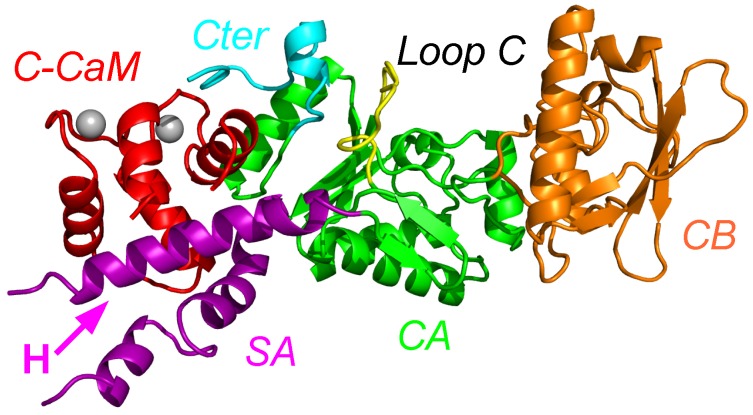
X-ray crystallographic structure (1YRT: [2]) of the complex AC/C-CaM. The AC domain includes three main subdomains, named CA (green), CB (orange), and switch A (SA) (purple). The switch A is named according to the three switches A, B and C, displaying large conformational changes [1] during the Edema Factor (EF) conformational transition. In AC, the regions corresponding to the EF switches were marked by Guo and coworkers [2]. The region corresponding to the switch C is the C terminal tail (cyan), and the one corresponding to the switch B is the catalytic loop (yellow). The two regions are included in the domain CA. The residue definitions of the regions are the following: residues 1–55, 181–191, 255–293 and 307–339 for CA excluding the C-terminal tail and the catalytic loop, residues 294–306 for the catalytic loop, residues 340–358 for the C terminal tail, residues 56–180 for CB, residues 192–254 for SA. These numbers are reduced by 6 for the residue numbers in 1YRT.

The X-ray crystallographic structure of the complex between AC and the N terminal lobe of calmodulin (C-CaM) was determined by Guo and coworkers [2] (Figure 1). Different sub-domains of AC have been described by these authors as: catalytic core A (CA), catalytic core B (CB), Switch A (SA), the catalytic loop C and the C terminal part of the structure (see the caption of Figure 1 for precise definitions). In the X-ray crystallographic structure of the AC/C-CaM complex, C-CaM interacts with AC through an interaction of the CaM EF-hand with the helix H, and through an interaction of the Ca${}^{2+}$ loop of C-CaM with the C terminal part of AC. By contrast, in the X-ray crystallographic structure of EF/CaM [1], the two lobes of CaM interact with the helical domain of EF, which is not present in AC, and with the SA domain of EF, much smaller than the one of AC.

The interaction calmodulin/AC is quite different than the interaction calmodulin/EF. Indeed, the barrier of activation of AC is smaller than the one of EF, as the affinity of AC for calmodulin (CaM) is about 0.2 nM [15], whereas it is 20 nM for EF [2,16]. Initial studies of AC/CaM interaction suggested that the most important aspect of the interaction between CaM and AC is the interaction between CaM and the α helix H from AC. Indeed, mutations of Methionines, which are in direct interaction with the helix H, induce a strong reduction of the AC affinity for CaM [17]. In addition, the α helix H can be considered as a structural anchor because the peptide spanning the sequence of helix H folds as an α-helix in solution [18,19]. Finally, the study of isolated AC by several biophysical approaches showed [20] that: (i) the isolated protein has a shape less elongated than AC in interaction with C-CaM; (ii) the percentage of α helices is reduced in the free AC; (iii) the free AC is more hydrated. One should notice that the study of Karst et al. [20] was performed using an intact CaM in contrast to the X-ray crystallographic structure determined in the presence of C-CaM [2].

## 2. Interaction between Calmodulin and AC

Molecular dynamics studies of AC started in 2009 with recording a series of trajectories [21] on free AC, on AC in complex with Ca${}^{2+}$-loaded C-CaM and in complex with apo C-CaM. This study showed that the calcium ions and the C-terminal lobe of calmodulin (C-CaM) play distinct roles in the interaction with AC. Indeed, the removal of calcium ions from C-CaM increases the AC flexibility, but the removal of C-CaM induces a dramatic drift of the AC conformation.

Isolated AC conformations show a general tendency to become less elongated, as the two protein extremities (regions SA and CB) tend to get closer [21]. This conformational tendency is in agreement with the observations made by Karst et al. [20] that the isolated AC display a shape closer to a sphere than the AC in interaction with C-CaM [2]. The conformational change of AC is far from negligible as the protein gyration radius oscillates within the range 24–27 Å. Nevertheless, the conformational drift of AC corresponds to oscillations around the X-ray crystallographic structure [2], and no conformational transition to a new basin is observed for the protein. The AC conformation observed in X-ray crystallographic structure is thus metastable at the nanosecond timescale.

At the extremity of the SA region, the loop containing residues 226–232 is not visible in the crystal, which is the sign of a large conformational heterogeneity. This loop has been reconstructed using Modeller [22] and displays similarly large internal mobility along the molecular dynamics (MD) trajectories. The other parts of the SA region also display more mobility if the ions Ca${}^{2+}$ are removed, and even more in the isolated protein. Within the SA region, large variations of relative orientations between α helices produce major conformational variability.

The calmodulin conformation has been extensively studied [23]. One major conformational motion of this protein is the motion moving the two lobes with respect to each other. However, another smaller motion has been described if the calcium ions are removed from the protein: the closing of the EF-hands [24,25], which are the angles between the α helices of the hands, slightly decrease.

The analysis of C-CaM conformation reveals that this lobe of calmodulin is in the open conformation, with α helices being perpendicular. Contrary to the observations on isolated calmodulin [24,25], this conformation is kept in the presence as well as in the absence of ions Ca${}^{2+}$. The interaction with AC thus forces C-CaM to stay in an open conformationno matter the level of calcium ions [21]. The Methionine residues 109, 124 and 145, which belong to the CaM hydrophobic patch (I85, A88, V91, F92, L105, M109, L112, L116, M124, F141, M144, M145, and A147) [26], are in close interaction with the α helix H of AC. This agrees with the previous experimental observation [17] that the interaction of CaM with AC depends on the reduced state of these Methionines. In addition, the α helix H is the helix displaying the most stable structure within SA along the MD trajectories, in agreement with the NMR study of this isolated peptide in solution [18,19].

An original approach has been developed [27] for describing the interactions between protein complexes formed involving several sub-domains. This approach intends to produce coarse-grained models of the energetic features of a given complex. To overcome the bias introduced by the empirical methods [28] for calculating interactions, all interaction energy values between the protein sub-domains are sorted and this order is compared to orders obtained when each sub-domain is removed from the complex. The influence of a given sub-domain is then estimated by the variation of order.

**Figure 2:**
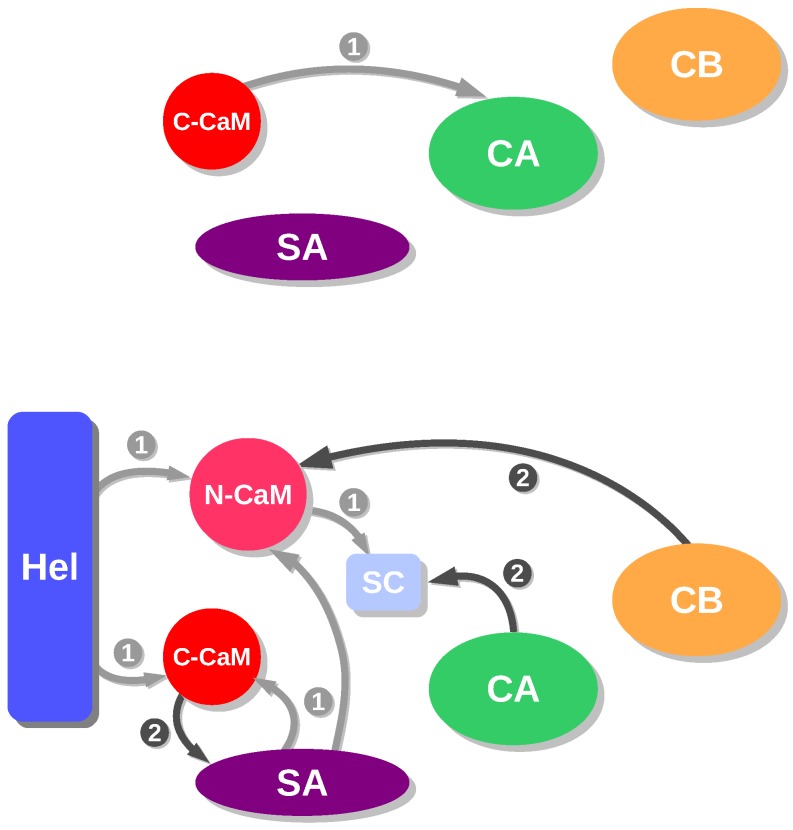
Schemes of energetic influences for AC/C-CaM (top) and EF/CaM (bottom) in the presence of Ca${}^{2+}$ ions. In the complex AC/C-CaM, the different regions are defined as in the caption of Figure 1. In the complex EF/CaM, the different regions are defined in the following way: CA residues 292–345 and 490–501 and 576–622), CB (residues 346–489), SA (residues 502–575), Hel (residues 660–767), SwitchC (residues 623–659), N-CaM (N terminal lobe of calmodulin: residues 5–84), C-CaM (C terminal lobe of calmodulin: residues 85–147). This figure was reproduced from [21].

The analysis of energetic influences is a coarse-grained analysis model that was proposed to describe the hierarchy in the assembly of macromolecules [29]. This approach was adapted [30] to the case of EF/CaM and AC/C-CaM complexes, by using the division of each partner in regions determined from the X-ray crystallographic structures and MD trajectories, and produced diagrams describing the interactions with the complexes EF/CaM and AC/C-CaM from the frames recorded along a MD trajectory [21]. The diagrams obtained for the EF/CaM and AC/C-CaM complexes display quite different qualitative features (Figure 2), as many more influences and intricate patterns are observed for EF/CaM than for AC/C-CaM. This topological feature is not surprising, as the interaction between EF and CaM is required to move apart the EF helical domain and the CA domain in order to insert CaM. On the contrary, the α helix H, to which C-CaM is in direct interaction, is more accessible to AC binding. This difference in the scheme of energetic influences for AC and EF agrees also with the higher affinity to CaM observed for AC than for EF [2,15,16].

**Figure 3:**
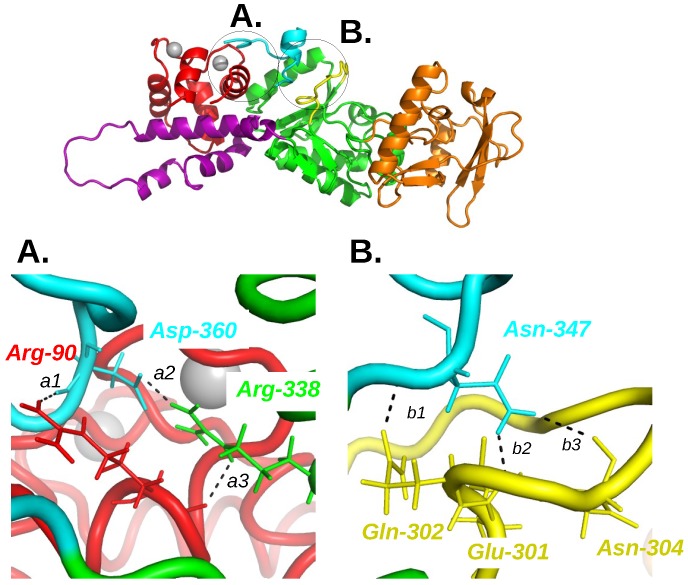
Overview of the AC/C-CaM complex structure (1YRT) drawn in cartoon with zooms **A** and **B** at the interface between C-CaM, C terminal tail and catalytic loop. The C-CaM lobe is colored in red, the C terminal tail in cyan, the catalytic loop in yellow, the remaining part of CA in green, the region CB in orange and the ions Ca${}^{2+}$ are drawn as silver spheres. The residues involved in hydrogen bonds in the presence of ions Ca${}^{2+}$ and for which the hydrogen bonds are disrupted in the absence of ions Ca${}^{2+}$ and AC are drawn in sticks and labeled in color according to the complex domain to which they belong. The corresponding hydrogen bonds are labeled *a1*, *a2*, *a3* for the interaction C-CaM/C terminal tail/CA and *b1*, *b2*, *b3* for the interaction C terminal tail/catalytic loop. This figure was reproduced from [21].

In the energetic influence scheme of the complex AC/C-CaM, the single influence observed from C-CaM to the region CA of the AC domain, inspired the authors to look at the variation of hydrogen bonds involving CA and C-CaM residues if the calcium ion are removed (Figure 3). The removal of calcium ions induced the breaking of hydrogen bonds involving residues D360, R338 and N347 located in the C-terminal extremity of AC, and R90 located in an α helix of the EF hand 3 in C-CaM.

Independent experimental studies of calmodulin [31] reveal that the interaction of calmodulin with AC increases the apparent Ca${}^{2+}$ binding in C-CaM. This is a close relationship to the observations of Selwa et al. [21], as the involvement of R90 in hydrogen bond with AC and the displacement of CaM α helices due to opening and closure of EF-hands [24,25] are directly connected.

The three following hydrogen bonds are broken during the MD trajectories [21] when the Ca${}^{2+}$ ions are removed: (i) between R90 of C-CaM and D360; (ii) between R338 and D360 within the CA region in AC; (iii) between N347 and the catalytic loop of AC. The residues involved being located in the C-loop (Q302, E301 and N304) and in the C-terminal extremity (R338, D360 and N347), the hydrogen bonds connecting them and C-CaM, draw a network from C-CaM to the AC catalytic site and are thus directly related to the AC function. The importance of these residues was confirmed by mutagenesis studies [32], which shows that mutating these residues in Alanines induces a decrease of the affinity of AC for CaM. As the simultaneous mutations of the three residues is required to significantly reduce this affinity, it seems that the residues act cooperatively, which agrees with a model of the network transmitting the conformational transition from CaM to the AC catalytic site.

The initial model of AC/CaM interaction was focusing on the interaction between CaM and the helix H and presents the feature to be potentially less specific, as CaM interacts with a huge variety of peptides [33] through its hydrophobic patches [26]. The molecular dynamics studies reviewed here revealed a different C-CaM/AC interaction picture where C-CaM stabilizes AC by a steric hindrance on the conformational drift of SA, whereas the Ca${}^{2+}$ ions allow further stabilization by the establishment of a hydrogen bond network extending from C-CaM to the AC catalytic loop. The C-terminal extremity of AC seems thus to play a more important role than previously thought. In addition, the elongated conformation of AC observed in the high resolution structure of the complex AC/C-CaM presents metastability, which is the sign of a significant energy barrier present between AC in complex with CaM and other regions of the AC conformational landscape. Due to this metastability, it was relevant to use enhanced sampling approaches to explore the AC conformational landscape.

## 3. Conformational Landscape of Free AC

**Figure 4:**
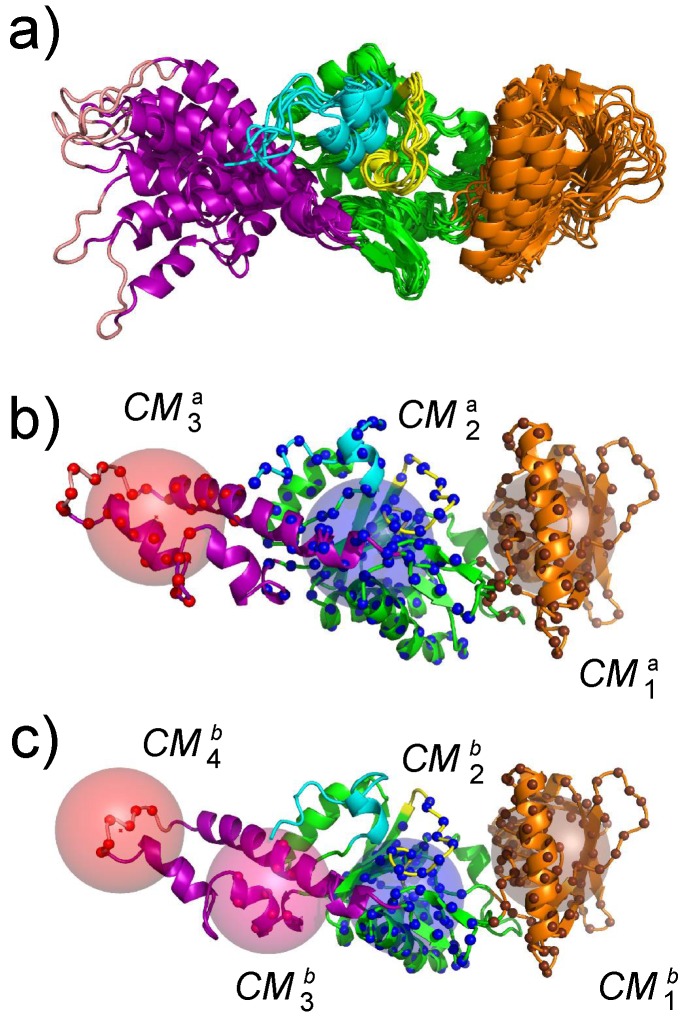
(**a**) superposition of representative conformations sampled along a molecular dynamics (MD) trajectory. (**b**,**c**) definition of collective variables used during the temperature accelerated molecular dynamics (TAMD) and soft ratcheting TAMD (sr-TAMD) trajectories on AC. The collective variables are represented as colored spheres centered on three (**b**) or four (**c**) geometric centers of AC regions in which the atoms Cα are drawn as balls with the same color as the corresponding sphere. This figure was reproduced from [32].

The first step for realizing an exploration of the AC conformational space using an enhanced sampling approach was the determination of collective variables. These collective variables are geometrical parameters that describe the variation of protein conformation during the conformational space exploration. Geometric centers were used on AC. These centers have been extracted using the PsiQRD approach [34]: sub-domains of the protein structures have been determined, in order that the relative motion of these sub-domains considered as rigid would permit reconstruction of the internal mobility observed along a MD trajectory. Sets of three and four geometric centers have been used, located in CB, CA, SA for the three centers, and in CB, CA, and the two parts of SA for the four centers (Figure 4).

The enhanced sampling approach used was the temperature accelerated molecular dynamics (TAMD) [35,36]. This approach is based on the coupled evolution of two trajectories, the first trajectory being the usual MD trajectory including terms restraining the collective variables to target values, and the second evolution equation describing the evolution of the targets values of the collective variables. In the two evolution equations, the effect of temperature is modeled using a Langevin thermostat, with a usual temperature around 300 K for the first equation and a larger temperature of about thousands or ten thousands of degrees Kelvin for the second equation. The large friction used for the Langevin thermostat at high temperature and the large force constant of the collective variable restraints guarantee the stability of the system and that its evolution is adiabatic. The use of the large temperature in the equation describing the evolution of the target values produces a decrease of the energy barriers on the free energy surface, thus permitting a better exploration of the conformational space.

**Figure 5:**
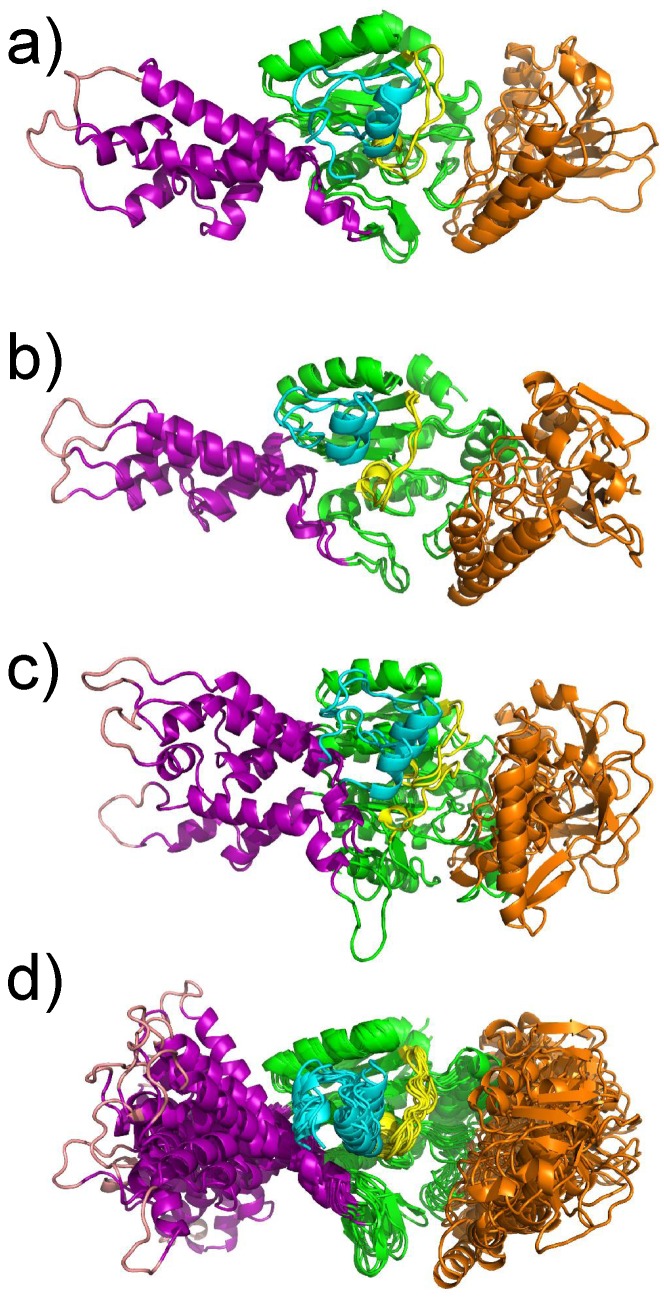
Representative conformations extracted using the Lyman–Zuckerman algorithm [37] with a cutoff d${}_{C}$ = 2.7 Å from the (**a**) MD and (**b**–**d**) different TAMD trajectories. The protein regions are colored in purple (SA), green (CA), cyan (C terminal), yellow (catalytic loop) and orange (CB). The representative conformations were transformed by rigid roto-translation to get their regions CA superimposed to each other. This figure was reproduced from [32].

The effect of the TAMD on the catalytic domain of AC is an increase of amplitude of the oscillation of the SA and CB domains corresponding to an amplification of the motion observed in the MD trajectory of isolated AC [21]. The motions along TAMD trajectories (Figure 5) agree with the less elongated structure experimentally observed for the isolated protein. The decrease of secondary structure elements is also in agreement with Karst et al. [20]. Nevertheless, the protein structure does not undergo a transition, pushing it far from the basin of the X-ray crystallographic structure, but rather oscillating around the X-ray structure. This oscillation may be explained by a more fluid structure of the water molecules around the protein, and can be due to a conformational entropic barrier preventing the protein from making a transition from the X-ray structure basin. This barrier may also arise from an inappropriate choice of the collective variables, which pushes the system toward a free energy barrier. The detection of a water molecule bridging residues from the domains SA and CA made possible obtaining one less elongated conformation, which is relatively stable along several tenths of ns of MD trajectories [32].

In order to induce a more efficient exploration of the conformational landscape, a Metropolis criterion was added to the evolution equation of the target values for collective variables [38]. This criterion permits accepting or rejecting proposed target values according to the molecular global shape of the protein induced by these values. Indeed, if the geometric centers are organized to correspond to a more globular shape, they are accepted. On the other hand, target values of the geometric centers giving rise to a more elongated shape are rejected with a certain probability. This Metropolis criterion was introduced in the literature as the soft-ratcheting criterion [39,40], and the corresponding TAMD was thus called sr-TAMD, sr standing for “soft-ratchetin”.

**Figure 6:**
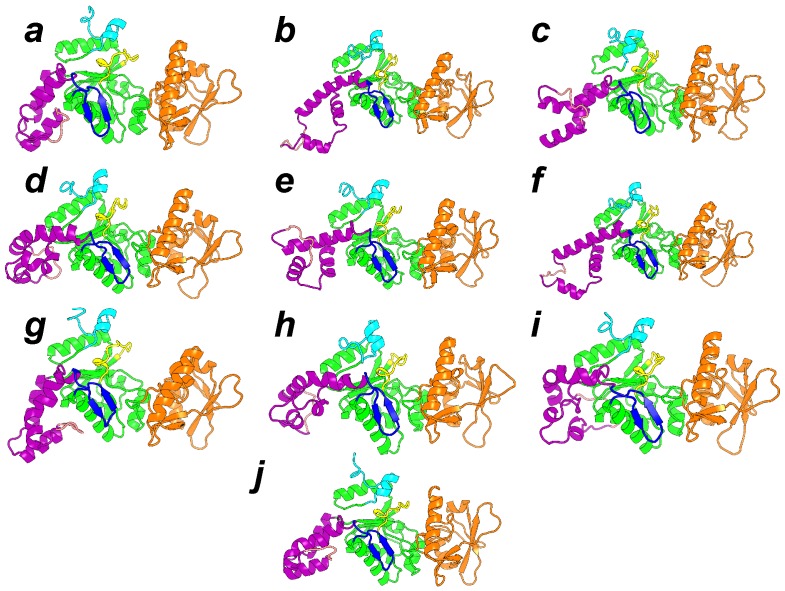
Representative conformations of the 10 clusters, labeled from *a* to *j*, and detected along sr-TAMD and MD trajectories. The conformation labels corresponding to the labels written on the SOM figure. Each conformation is drawn in cartons, with the region CA in green, the region CB in orange and the region SA in magenta. The catalytic loop is colored in yellow, and the C terminal loop in cyan. The β-hairpin region (residues 259–273) is colored in blue. This figure was reproduced from [38].

**Figure 7:**
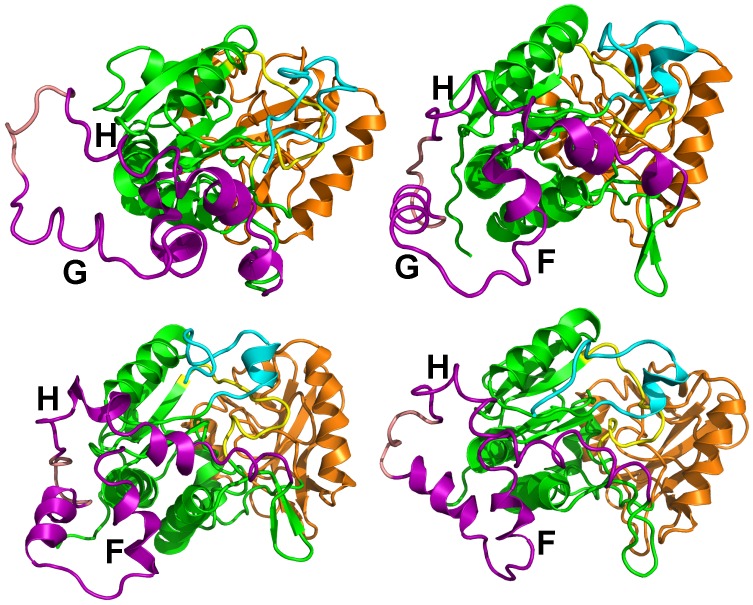
Examples of conformations sampled during sr-TAMD trajectories and displaying destabilized α helices in the SA region. These simulations were recorded on the AC364, which contains six additional residues at the N-terminal extremity with respect to the AC sequence in the X-ray crystallographic structure [2].

The sr-TAMD approach allowed to obtain a series of AC conformations [38], displaying a significantly less elongated shape than the starting X-ray crystallographic structure, as well as the conformations sampled in the previous TAMD (Figure 6). Nevertheless, one should keep in mind that a side-effect of the geometric centers is to rigidify the group of atoms on which they are defined. The AC conformations obtained by sr-TAMD have thus the tendency to display well-formed α helices, although it is also possible to detect conformations with destabilized α helices (Figure 7). Due to the destabilization of the α helices, these conformations are prone to be more hydrated than in the X-ray crystallographic structure of AC, in agreement with the observations made by Karst et al. [20].

The sets of representative conformations of AC (Figure 6) obtained using the sr-TAMD approach provide information on AC which can be compared with experimental measurements possible on this protein. The information obtained using various biophysical techniques [41,42] could be used to sort out the conformations proposed by sr-TAMD in order to determine information at the atomic level on the inactive state of AC.

The most common feature of the set of representative conformations describing the AC inactive state (Figure 6) is a large reorganization of the α helices in the SA domain. This reorganization permits a reduction in the elongated shape of AC, and is in contrast to the minimal variation observed for CA and CB. The large variation of the SA architecture agrees with the experimental observation [20] that the largest secondary structure variation between active and inactive AC is observed in SA. One should also remark that in the set of representative conformations (Figure 6), the SA domain moves apart from the C-terminal extremity, keeping it accessible. The mutated residues observed in the C terminal region [43] are thus available for interaction with CaM. The β hairpin (residues 259–273), colored in blue in Figure 6, is in most of the conformations less accessible to the solvent, due to the vicinity of the region SA. As this hairpin was shown [44] to play an important role in the interaction with the N-terminal lobe of CaM (N-CaM), its reduced accessibility certainly has an impact on the interaction between AC and intact calmodulin, and might indicate that the interaction AC/C-CaM is established before the interaction AC/N-CaM.

The future perspectives for the exploration of the AC conformational landscape will be exploiting the experimental measurements obtained in the inactive state to evaluate the obtained conformations as well as to orient the search of the conformational landscape. Indeed, the use of the soft-ratcheting criterion, in the frame of sr-TAMD, provides a large variety of possible ways to orient the search. Once a good agreement will be found between the proposed set of representative conformations and the experimental observations on the inactive state, AC engineering can be performed to modify the populations of AC conformations in the inactive state, and to fine-tune the AC activation using effectors different from calmodulin. Furthermore, a better knowledge of the AC inactive state could be used for discovering inhibitors of this protein.

## 4. Searching Inhibitors of AC Activation

**Figure 8:**
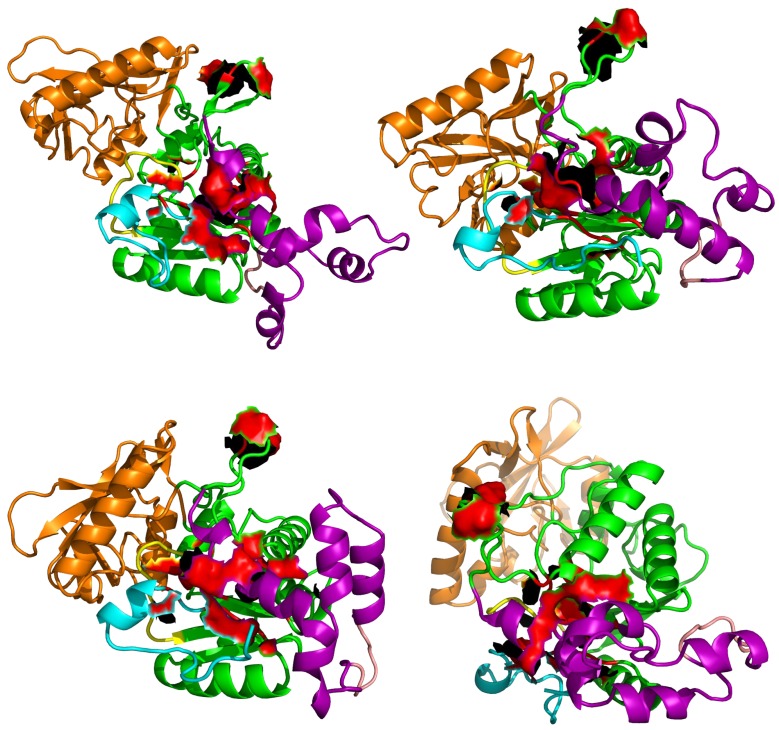
Position of residues of AC aligned to the EF residues forming the pocket SABC, in various representative conformations sampled along sr-TAMD trajectories recorded on AC364. The residues aligned to the EF residues are displayed as red surface.

The drug design approach is not a priority for the protein AC, as the whooping cough is still sensitive to antibiotic treatments. However, the development of resistance in *Bordetella pertussis* [10,11] will make more important in the future the search of inhibitors. A previous molecular modeling study of the adenyl cyclase EF from *Bacillus anthracis* has led [45] to the discovery of EF inhibitors by targeting the EF pocket SABC displaying variation of shape between the active and inactive states of EF. Surprisingly, these EF inhibitors also displayed an activity against AC [45], which was discovered fortuitously when AC was used as a control to avoid the detection of promiscuous ligands. As a prospective argument, the residues of the pocket targeted on EF are also present in AC [45]. The position of these residues in some AC conformations sampled along sr-TAMD trajectories are shown (Figure 8) and reveal that some of the residues can form pockets. The presence of such pockets supports the interactions experimentally observed between AC364 and the thiophen ureoacids.

The similarity between EF and AC is also supported by the sequence alignment of proteins of the MARTX (Multifunctional-Auto-processing Repeats-in-ToXin) toxin family [46], including EF, AC and ExoY. Although the sequence similarity is not very high, the alignment, coupled with the possible similarity of the inhibitors interaction in EF and AC, can be exploited to push forward the structural, biophysical and biochemical knowledge on the proteins MARTX.

## 5. Conclusions

Starting from the limited knowledge of the AC conformational landscape, an extensive use of the tools of molecular modeling was performed for seven years. The concomitant use of several bioinformatics approaches: molecular dynamics simulations, of enhanced sampling approaches and virtual screening has permitted obtaining additional information on the structural and dynamics AC behavior. Based on this information, the model of interaction AC/CaM has evolved from a focus on the interaction between CaM and α helix H to a double interface model: an interface being established between the C-CaM hydrophobic patch and the helix H and the other interface between the C-CaM Ca${}^{2+}$ loop and the C-terminal region of AC. The second interface has a possible connection to the AC catalytic site, which gives structural biology arguments to relate the interaction with CaM to the AC function. Then, the exploration of the conformational landscape of the isolated AC, using enhanced sampling techniques, allowed for proposing a description of the conformational landscape of inactive AC. This description can be confronted to biophysical experiments on the AC inactive state, to provide information at the atomic level on the AC conformations populated in this state. Finally, virtual screening techniques, already applied with success to the adenyl cyclase EF from *Bacillus anthracis*, could be used in AC as well as in other members of the family of MARTX proteins, in order to improve the structural, biophysical and biochemical knowledge of these proteins.
